# Comparative Analysis of Splicing Alterations in Three Muscular Dystrophies

**DOI:** 10.3390/biomedicines13030606

**Published:** 2025-03-01

**Authors:** Vanessa Todorow, Stefan Hintze, Benedikt Schoser, Peter Meinke

**Affiliations:** 1 Friedrich-Baur-Institute, Department of Neurology, LMU Klinikum, Ludwig-Maximilians-University Munich, 80336 Munich, Germany; 2 Department of Molecular Biophysics and Biochemistry, Yale University, New Haven, CT 06511, USA

**Keywords:** myotonic dystrophy type 1, facioscapulohumeral muscular dystrophy, Emery–Dreifuss muscular dystrophy, splicing, myopathy, missplicing, splicing profile

## Abstract

**Background/Objectives**: Missplicing caused by toxic *DMPK*-mRNA is described as a hallmark of myotonic dystrophy type 1 (DM1). Yet, there is an expressional misregulation of additional splicing factors described in DM1, and missplicing has been observed in other myopathies. Here, we compare the expressional misregulation of splicing factors and the resulting splicing profiles between three different hereditary myopathies. **Methods**: We used publicly available RNA-sequencing datasets for the three muscular dystrophies—DM1, facioscapulohumeral muscular dystrophy (FSHD) and Emery–Dreifuss muscular dystrophy (EDMD)—to compare the splicing factor expression and missplicing genome-wide using DESeq2 and MAJIQ. **Results**: Upregulation of alternative splicing factors and downregulation of constitutive splicing factors were detected for all three myopathies, but to different degrees. Correspondingly, the missplicing events were mostly alternative exon usage and skipping events. In DM1, most events were alternative exon usage and intron retention, while exon skipping was prevalent in FSHD, with EDMD being in between the two other myopathies in terms of splice factor regulation as well as missplicing. Accordingly, the missplicing events were only partially shared between these three myopathies, sometimes with the same locus being spliced differently. **Conclusions**: This indicates a combination of primary (toxic RNA) and more downstream effects (splicing factor expression) resulting in the DM1 missplicing phenotype. Furthermore, this analysis allows the distinction between disease-specific missplicing and general myopathic splicing alteration to be used as biomarkers.

## 1. Introduction

On the molecular side, there are often multiple pathways affected under disease conditions. While these may be secondary effects, it is still necessary to understand the entanglement of this misregulation, whether it be to identify targets for therapeutic interventions aiming to attenuate clinical symptoms or as readouts for disease progression and therapeutic success. Muscular dystrophies are inherited myopathies characterized by progressive muscle weakness and wasting of variable distribution and severity [1]. Recently, we could identify the misregulations of splicing factors in three different muscular dystrophies: myotonic dystrophy type 1 (DM1), facioscapulohumeral muscular dystrophy (FSHD) and Emery–Dreifuss muscular dystrophy (EDMD), based on RNA sequencing [2,3,4]. While there are striking similarities between them, each disease features its own splicing patterning, and its examination might help us understand not only the complex molecular pathology underlying these diseases, but also the fundamental principles of splicing regulation.

DM1 is a multisystemic disorder, with the skeletal muscle and brain being the primarily affected organs. The list of clinical symptoms includes myotonia, skeletal muscle weakness and wasting, cardiac arrhythmia, cataracts, insulin resistance and endocrine dysfunction [5,6,7]. Typically, the distal muscles are affected more severely, often the finger and wrist flexors and the foot extensors [5]. The causative genetic defect for DM1 is a pathological CTG-repeat expansion in the 3’UTR of the *DMPK* (dystrophia myotonia protein kinase) gene [8]. This repeat expansion is somatically unstable depending on the age and length of the expansion [9,10], which leads to tissue mosaics [11]. More than 50 CTG repeats are considered to be causative [12]. The repeat length correlates to some extent with the severity and time of manifestation of the disease [13,14]. Several different mechanisms have been described that can potentially contribute to the expression of the clinical symptoms of DM1. These include the alternative splicing of several mRNAs [15,16,17,18], miRNA misregulation [19,20,21,22,23,24,25], altered transcriptional regulation [19,26,27], inhibited translation [28,29], alternative polyadenylation of several mRNAs [30] and repeat-associated non-ATG (RAN) translation [31,32,33]. While it is likely that these mechanisms are not exclusive, but combine to lead to the expression of the complex phenotype, the favorite and probably most intensively investigated mechanism is alternative splicing, which is caused by the formation of hairpin structures in the extended CUG repeat containing *DMPK* RNA transcripts [34]. These hairpin structures sequester several RNA-binding proteins, including muscle-blind proteins (MBNL1-3) [35,36]. The resulting nucleoplasmic depletion of MBNLs and the parallel gain of function of CELF1 by hyperphosphorylation [37,38] result in a misbalance of splicing and a shift towards an embryonic splicing pattern. Prominent misspliced genes are *CLCN1* [39,40] and *DMD* [41], amongst others.

EDMD can be described as a disease of the nuclear envelope (NE), since all mutated genes encode NE proteins or at least an NE-localized isoform [42]. So far, the following genes have been identified: *LMNA* [43,44], *EMD* [45], *FHL1* [46,47], *SYNE1/2* [48], *SUN1* [49], *PLPP7* and *TMEM38A* [50]. Two main theories try to explain the disease pathology. These are the so-called “structural” hypothesis, proposing a weakening of the NE and cytoskeleton connection and resulting in an increased susceptibility to mechanical stress—especially in cells suffering an intense mechanical strain—and the second one, the “gene expression” hypothesis, which proposes an altered chromatin organization resulting in changed gene expression patterns affecting specific tissues [51,52]. The identification of *PLPP7* and *TMEM38A* mutations in EDMD patients supports the “gene expression” hypothesis, as both genes encode muscle-specific NE proteins involved in genome organization, and the respective mutations alter the gene positioning [50]. Clinically, EDMD has been initially described as a triad of early contractures of the elbows, Achilles tendons and cervical muscles; slow progressive muscle wasting and weakness with a humeroperoneal distribution in the early stages; and a cardiomyopathy usually presenting as heart block [53]. However, this holds mostly true for the X-linked form of EDMD caused by mutations in the *EMD* gene, while this is not so clear for the other forms of EDMD, especially the autosomal dominant forms. Therefore, Alan Emery suggested using the term “Emery–Dreifuss syndrome” [54].

While a repeat expansion causes DM1, the genetic cause for FSHD is a repeat contraction. Healthy individuals carry 11 to 100 or more D4Z4 repeats, each containing a *DUX4* gene, located on chromosome 4q35. A contraction to 10 or less of these D4Z4 repeats is the genetic cause of FSHD [55]. This region is usually associated with heterochromatin, and the *DUX4* gene, a homeobox transcription factor [55,56], is not expressed in healthy individuals. However, the combination of the 4qA haplotype, which contains a polyadenylation signal, and the repeat contraction results in a loss of *DUX4* repression. The DUX4 protein is thought to trigger signalling cascades by activating other transcription factors and hundreds of genes, resulting in rapid cell death [57]. Clinically, FSHD typically starts with an initial asymmetric weakness and atrophy of the facial, shoulder girdle and upper arm muscles, followed by a descending involvement of the distal lower extremities [58,59,60].

Although these three myopathies have distinct genetic origins and molecular pathologies, they all exhibit prominent muscle wasting and weakness. In this study, we compare the misregulation of splicing factor expression and the resulting splicing patterns. This aims at answering the question of whether these are just secondary downstream effects, or if there are disease-specific patterns hinting at more early pathological effects.

## 2. Materials and Methods

**Figure 1:**
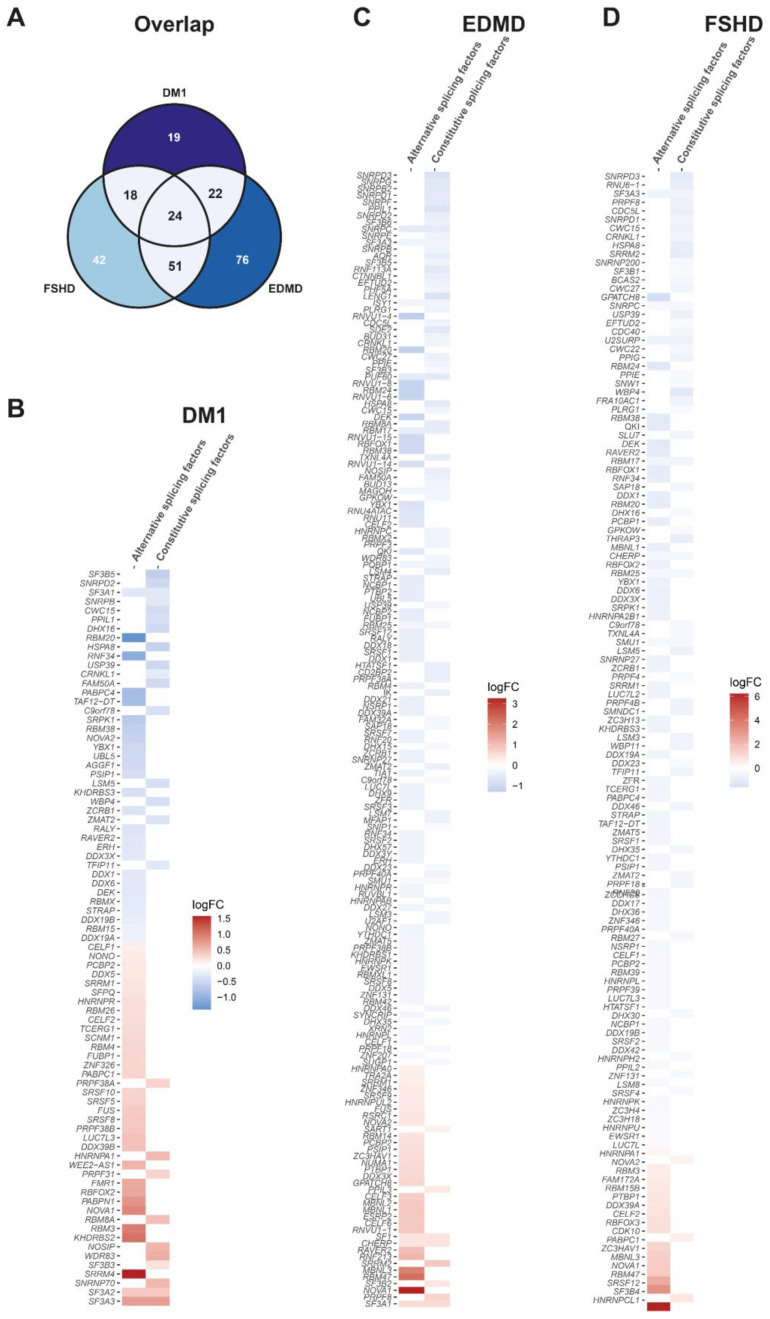
**Splicing factor gene expression.** (**A**) Overlap of differential expressed splicing factors between DM1, EDMD and FSHD and heatmaps of splicing factor gene expression (transcriptional level) in (**B**) DM1, (**C**) EDMD and (**D**) FSHD.

Published and peer-reviewed datasets were used for this analysis. For FSHD, a sequencing dataset containing MRI-informed muscle biopsies from the lower extremities of 36 individuals with FSHD was used [61]. The DM1 dataset was taken from a publication containing 120 RNASeq transcriptomes from skeletal and heart muscles derived from healthy and DM1 individuals [62]. The EDMD data come from a set containing 10 EDMD-derived differentiated muscle cell cultures [4]. The raw data from Wang et al. [61], de las Heras et al. [4] and Wang et al. [62] were trimmed using trimgalore [63] if necessary, mapped to the human genome assembly GRCh38 (hg38) and sorted by coordinates using STAR 2.7.9a [64] for analysis in DESeq2 and MAJIQ. The data quality was assessed using FASTQC. The expression analysis was conducted in R 4.0.4 using DESeq2 [65], and the splicing analysis was performed using MAJIQ [66]. The reads were counted using FeatureCount and analyzed following the standard DESeq2 workflow. The significance threshold (p-adjusted) was set to 0.05. For the expression analysis of splicing factors, custom-made lists of splicing factors, divided into alternative and core spliceosome using the spliceosome database (http://spliceosomedb.ucsc.edu/, accessed on 1 June 2023) [67] as well as splicing related GO terms, were created (Supplementary Table S1). For the heatmaps, only the significant genes were plotted. For the Venn diagram, the genes (Supplementary Table S2) were overlapped between the diseases (Figure 1). The index files for each aligned sample were generated using bamtools, and the splicing analysis was performed using MAJIQ (v2.3) in python (v3.9; https://www.python.org/) and visualized using Voila (v2.3; https://majiq.biociphers.org/).

**Figure 2:**
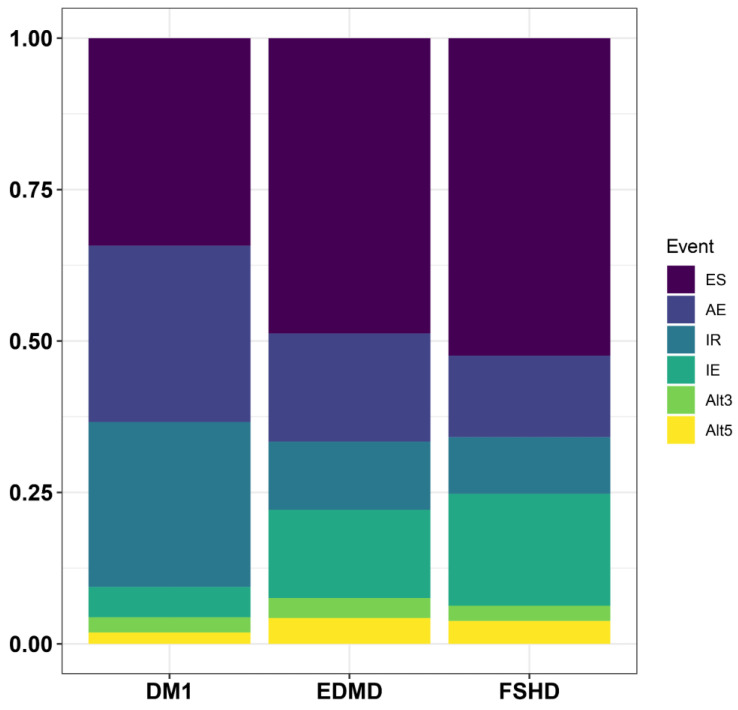
**Alternative splicing event distribution in DM1, EDMD and FSHD.** Event type indicated at right; ES: exon skipping, AE: alternative exon usage, IR: intron retention, IE: intron exclusion, Alt3: alternative 3′ SS, Alt5: alternative 5′ SS.

The splicing changes measured as psi (percent spliced in)-values of more than 0.1 or less than −0.1 were considered to be alternatively used between disease and control. For Figure 2, the MAJIQ output produced by the voila modulize function for the selected event types (exon skipping, intron retention, alternative 3′ splice site and alternative 5′ splice site) was filtered after the psi-values, FDR (>95%) and unique LSVs (local splicing variations) using the lsv_id variable (MAJIQ often, but not always, describes the same event from the perspective of different reference exons, so to avoid the overrepresentation of these events, only one was used). The distribution of the event types was then visualized in stacked barplots (ggplot2) for each event type.

**Figure 3:**
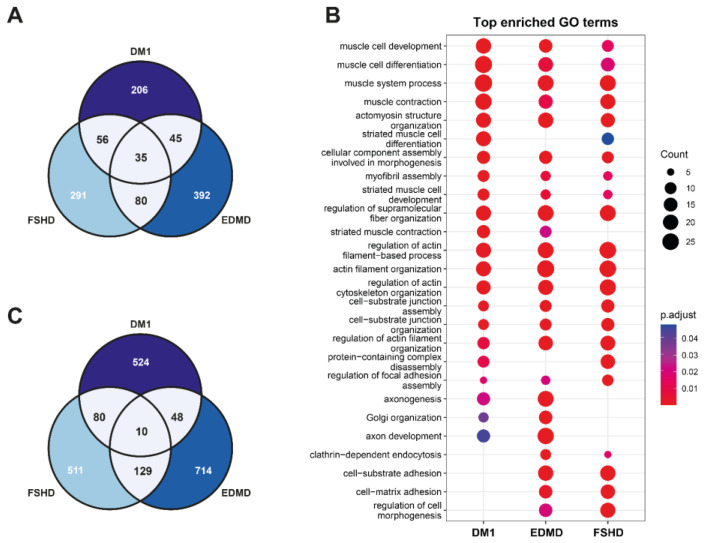
**Comparison of misspliced genes and splicing events in DM1, EDMD and FSHD.** (**A**) Overlap of genes misspliced in respective dystrophies. (**B**) Overlap of specific splicing events (LSVs). (**C**) Comparison of GO term enrichment analysis between dystrophies. Size of dots corresponds to number of genes; color corresponds to adjusted *p*-value of enrichment (see legends).

**Figure 4:**
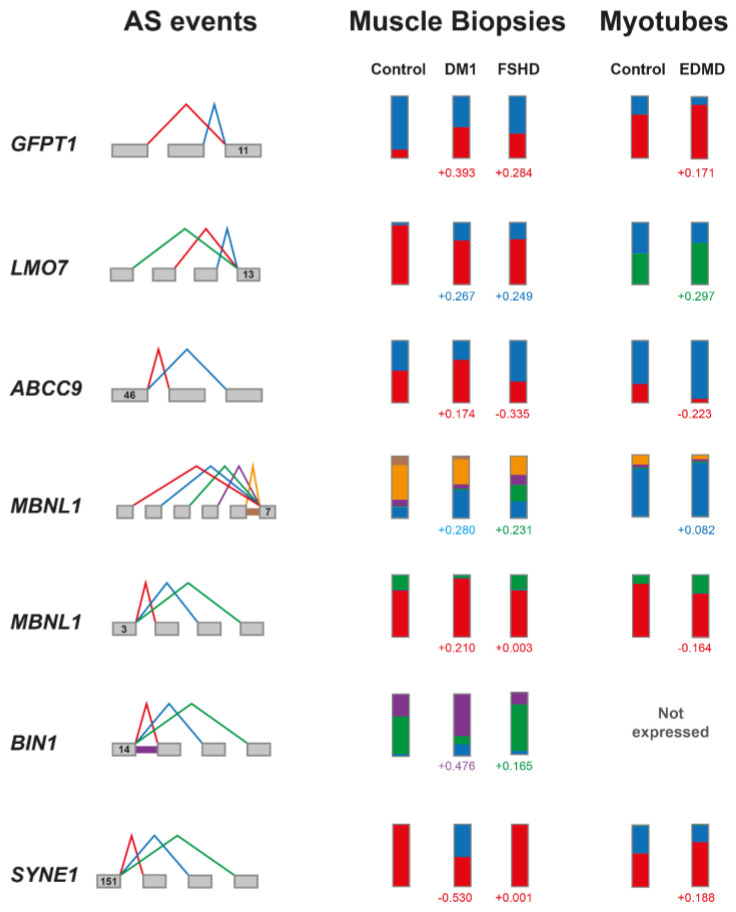
**Splicing events in DM1, EDMD and FSHD.** The exons are indicated as grey boxes, and the splicing events are displayed as colored lines (**left**). The percentages of splice event usage are shown in the bars for the control muscle biopsies and control myotubes, as well as the DM1 and FSHD biopsies and EDMD myotubes (**right**: the full height of each bar represents 100%, and the colored parts show the proportions of the respective splice events). The PSI-values are below the bars, and the color corresponds to the event (**left**) in the same color.

The misspliced genes, as well as the LSVs, were overlapped between the diseases (Venn diagrams in Figure 3), and the unique genes were used for a comparative GO term analysis using gprofiler2 and enrichplot. Of note, the lists for the overlap of both genes and LSVs were obtained from MAJIQ’s voila view command (thresholds were FDR > 95% and psi-values > 0.1 and <−0.1), so these events include all event types analyzed by MAJIQ (in contrast to Supplementary Tables S4–S6, in which only the selected event types are listed). Since the LSVs outputted by voila view are non-readable without MAJIQ itself, they are not attached as a supplementary table. If interested, please contact the corresponding author. Voila view was used for the visualization of specific events (Figure 4).

The following sample numbers were used for this analysis: DM1—11 patients (dorsiflexion strength 0–25% of normal values) and 11 controls (tibialis anterior muscle biopsies) [62]; FSHD—6 patients (assessed via MRI and expression of the biomarkers *LEUTX*, *KHDCL1*, *TRIM43* and *PRAMEF2*) and 9 controls (quadriceps muscle biopsies) [61]; EDMD—5 patients (gp1, patients with classic EDMD symptoms) and 2 controls (differentiated muscle cell cultures) [4].

## 3. Results

### 3.1. Splicing Factor Expression

To get an overview of how the genes encoding spliceosomal proteins are affected in all three diseases, we created a list of splicing factors divided into alternative splicing factors and constitutive splicing factors (Supplementary Table S1). As some proteins are described with multiple functions, this was preferred to a classic GO term enrichment analysis. This list was then used to compare the expression of the splicing factors in the DM1 [2] and FSHD patient muscle biopsies [3] and differentiated EDMD myotubes [4]. We decided in all three cases to use either the most severely clinically affected patient samples or, in the case of EDMD, the most severely molecularly affected group of samples. All disease samples were compared to their respective controls, and processed equally using the same bioinformatic packages and significance thresholds.

In the DM1 muscle biopsies, 83 genes encoding splicing factors are significantly differentially expressed compared to the healthy controls, with the log fold changes ranging between −1 and 1. In the FSHD muscle biopsies the number is 135, and in the EDMD myotubes 173, and the changes are also small, with a few exceptions like *NOVA1* (log2FC = +3 in FSHD) and *HNRNPCL1* (log2FC = +6 in EDMD). When comparing all splicing factors misregulated in each dystrophy (Supplementary Table S2), they share 24 (Figure 1A) including, among others, *NOVA1*/*2*, *SF3A3*, *CELF1* and *RBM20*. While in DM1, there appears to be a balance between the up- and downregulation of the splicing factors (Figure 1B), in EDMD and FSHD there is a general trend of downregulation of the splicing factors, with only a few alternative splicing factors being upregulated (Figure 1C,D). Of note, *MBNL1* is upregulated in EDMD, downregulated in FSHD and not significantly downregulated on the RNA level in DM1. Although the fold changes are mostly small, we hypothesized that a misregulation of many factors would still affect the splicing outcome of a subset of target genes, so we investigated and compared the differential splicing in all three diseases.

### 3.2. Splicing Profile

To investigate this, we first examined whether these changes in the splicing factor expression had an impact on the splicing profile, i.e., the splicing event distribution. All samples were analyzed using MAJIQ and filtered for an FDR > 95% and psi-values > 0.1 and <−0.1. In DM1, 342 genes were identified to be significantly misspliced, and in EDMD and FSHD 552 and 462, respectively. To visualize the splicing profile, the splicing alterations compared to the controls were quantified and grouped according to the type of event: exon skipping, alternative exon usage, intron retention, alternative 3′ splice site usage and alternative 5′ splice site usage. This revealed about 34% of the splicing alterations in DM1 to be exon skipping, while there was even more exon skipping in EDMD (~49%) and FSHD (~52%, Figure 2). Alternative exon usage was highest in DM1 (~29%), with fewer events in EDMD (~18%) and FSHD (~13%). Similarly, intron retention was highest in DM1, less in EDMD and least in FSHD (Figure 2). Given these profiles in combination with the above-mentioned expression changes, we hypothesize that broader downregulation of splicing as seen in EDMD and FSHD leads to more exon skipping, while more robust upregulation of splicing as seen in DM1 leads to preferential alternative exon inclusion. Detailed analyses have to be performed to test this, which exceeds the scope of this work.

### 3.3. Detailed Splicing Analysis

Next, we looked at the misspliced genes in more detail. In total, 35 genes are commonly misspliced among the dystrophies, and many more are shared by at least two of them (Figure 3A, Supplementary Table S3). Of note, the genes unique to each dystrophy belong to very similar pathways, namely muscle development, contraction and structure, neuromuscular junction (NMJ) formation and protein synthesis (Figure 3B). While this output is informative about the genes being affected by missplicing (altered splicing patterns compared to controls), this doesn’t give specific information about the actual specific splicing events (LSVs) within the genes. Looking at the specific LSVs, 10 splicing events are shared between all three dystrophies, and a remarkable 129 between EDMD and FSHD—even though myotubes and mature muscles have different splicing patterns (Figure 3C, description see Section 4). Notably, there are a few genes that are misspliced in DM1, and have been linked to a definitive symptomatic outcome, that are misspliced neither in FSHD nor EDMD, namely *CLCN1*, *ATP2A1/2* and *MYOM1*, of which the *CLCN1* missplicing results in the most unique feature of DM1: myotonia.

Among the 10 shared LSVs is *GFPT1*, an NMJ architectural protein-encoding gene, one of the best described misspliced genes in DM1 [68]. Notably, exon 10 skipping is increased in DM1, EDMD and FSHD (Figure 4, psi-values indicated in small numbers under bars). There are 138 LSVs that DM1 shares with EDMD, FSHD or both, and *GFPT1* is not the only well-established, “DM1-desribed” splice event: *BIN1* and *MBNL1* are misspliced at the same locus in FSHD, but with different outcomes. In *BIN1*, there is a preferential intron 14 retention in DM1, but a double exon skipping in FSHD, while in *MBNL1*, there is a triple exon skipping (exons 4, 5 and 6) in DM1, but a double exon skipping (exons 5 and 6) in FSHD. This is consistent with the general trend of increased exon skipping in FSHD compared to DM1. This is also demonstrated by exon 47 of *ABCC9* being rather included in DM1, but excluded in EDMD and FSHD. In the case of *MBNL1*, MAJIQ also identifies an exon 4 inclusion in DM1 (psi-value +0.21), while the controls have, in ~20% of the cases, a double exon skipping. DM1 thus moves towards the splice pattern of the control myotubes (about 10% inclusion and 90% double exon skipping), while the EDMD myotubes are closer to the mature muscle. FSHD has no significant change at this LSV. Similarly, the skipping of the muscle-specific exon DV23 (exon 152) in *SYNE1* in DM1 resembles the control myotubes, while the EDMD myotubes have a higher inclusion of DV23, and thus a shift towards adult muscle splicing (Figure 4).

## 4. Discussion

Although DM1 is seen as the splicing disease among the muscular dystrophies, we could show before that splicing is also affected in EDMD and FSHD, with many splicing factors misregulated and many genes misspliced which had not been shown or investigated in detail previously [2,3]. The missplicing in DM1 has been attributed to a MBNL/CELF disbalance caused by “toxic” *DMPK*-mRNA containing extended repeats [69,70,71]. We could, however, also identify the altered expression of splicing factors in DM1 [72]. This analysis aims to investigate the potential overlaps between the splicing alterations in DM1, EDMD and FSHD to understand the potential impact on the molecular pathology and the value as a biomarker.

When working on rare diseases with clinical variability, sample numbers are always a limitation. The prevalence of DM1, FSHD and EDMD is estimated at roughly 1:8000, 1:25,000 and 1:250,000, respectively [73,74]. While for the more frequent myopathies, DM1 and FSHD, there are publicly available sequencing data, even including information on how much the sequenced muscle is affected in some cases, this is not the case for EDMD. Thus, we used RNA-sequencing data generated from differentiated myotubes of EDMD patients and control primary muscle cell cultures generated before EDMD [4]. For DM1 and FSHD, we decided to use the patient groups with the most severely affected muscles by clinical evaluation for our analysis to overcome the problem of clinical variability [61,62]. As the sequencing data for each disease were generated using different protocols, we used controls generated in parallel to each sample group (using the same protocols) to analyze the expression and splicing changes for each group.

The expression levels of the splicing factors may contribute to the altered splicing profiles, provided that the changes in their expression lead to corresponding changes in protein levels and disrupt the required stoichiometry of the spliceosomes. Directly testing this hypothesis is challenging due to the limited availability of most samples. However, comparing splicing factor expression with splicing profiles across different diseases can offer insights into the potential effects. Our analysis reveals a general upregulation of alternative splicing factors across all three diseases, with the strongest increase observed in DM1, followed by EDMD and FSHD. Correspondingly, when the alternative splicing increases in all three diseases are compared to their respective controls, mirroring the upregulation of alternative splicing factors, the highest levels of alternative splicing are seen in DM1, followed by EDMD and FSHD.

Conversely, the constitutive splicing factors show a tendency toward downregulation, most pronounced in FSHD, followed by EDMD and DM1. Similarly, exon skipping is elevated in all three diseases, with the most pronounced effect in FSHD, followed by EDMD and DM1. This suggests that the stoichiometry of the available splicing factors directly influences the splicing outcomes. A deficiency in the core splicing factors essential for spliceosome assembly may cause the spliceosome to disassemble, leading to exon skipping. Likewise, if an alternative splicing factor required for splice site recognition is absent, the spliceosome formation at that exon may be impaired, also resulting in exon skipping. In contrast, an increased concentration of an alternative splicing factor could enhance the inclusion of its target exon. Alternative splicing rarely operates in a binary “spliced in” or “spliced out” manner, and even a 10% change is considered biologically significant. While our hypothesis remains untested, the findings presented here provide strong support for this mechanism. We encourage further research to explore this fundamental aspect of splicing regulation.

Our findings further indicate that on top of the sequestration of MBNL proteins and subsequent activation of CELF1, the misregulation of other splicing factors is a major factor influencing the splicing events in DM1. This is also well in line with the observed molecular pathologies in all three diseases, which lead to expressional changes. In EDMD, an altered genome organization affects the gene expression [50,51,52], while in FSHD, altered methylation has been proven to be a better predictive marker than the length of repeat contraction [75], and thus the gene expression is a relevant factor on top of the altered expression of the transcription factor DUX4 [55,56]. In DM1, not only are altered transcriptional regulation [19,26,27] and inhibited translation [28,29] described, but we can also show significant misregulation of the muscle-specific NE proteins involved in genome organization and a subsequent misregulation of the genes under their “positional control” [2].

While the resulting splicing factor misregulation and subsequent missplicing may be downstream of the initial pathomechanism, it is still important. For example, the missplicing of exon 10 in the *GFPT1* gene is a well-described biomarker for DM [68], but the same event is indeed present in all three myopathies analyzed here. It has been shown before by qPCR that many “DM1-specific” splice events are shared between DM1 and Duchenne muscular dystrophy (DMD) [76] including, among others, *MBNL1*, *ATP2A1*, *TTN*, *TNNT2* and *MEF2A/C*. These specific, shared events are very likely downstream events due to myopathic changes rather than primary events due to RNA-binding protein sequestration, and thus are not suitable for the prediction of disease progression. Instead, unique missplicing events, like in *CLCN1*, that are not found under non-DM conditions should be used as biomarkers, especially for therapeutic treatments [39,40].

We want to highlight the shared splicing events that have not been previously investigated in DM1. For example, the transcription factor *LMO7* displays an exon 12 inclusion in DM1 and FSHD. LMO7 has important muscle-specific functions and, interestingly, interacts with emerin at the NE [77]. Wang et al. found that exon 20 skipping in *LMO7* was among the most dysregulated splice events in DM1 in the heart, but not the skeletal muscle [62], highlighting an important function of this gene, but also that the exact splice event differs between tissues even in the same disease. The missplicing of transcription factors potentially adds to a cascade of gene expression changes and missplicing, thus complicating the classification of upstream and more downstream events in molecular pathology.

Another potentially interesting gene, *ABCC9*, encodes an ATP-dependent potassium channel in the skeletal and cardiac tissue, and thus plays important roles in innervation. In *ABCC9*, MAJIQ detects increased levels of exon 47 inclusion in DM1 (psi-value + 0.17) and exclusion in FSHD and EDMD (−0.34 and −0.22, respectively). This could be of interest, as there are, especially in DM1, indications for a denervation phenotype [78].

## 5. Conclusions

These findings raise an important question: how can we determine whether the splicing events observed in DM1 are a direct consequence of toxic RNA (i.e., a primary effect) rather than a secondary result of muscle damage, given that dystrophic muscles in EDMD, FSHD and DMD exhibit similar alternative splicing patterns? While a detailed comparative analysis of DM1-specific missplicing may help address this, the timing and origin of the splicing alterations in EDMD and FSHD remain uncertain. Are these changes a cause or a consequence of muscle atrophy? Or do they serve as early contributors to disease progression? If these splicing alterations represent a shared cascade of events in the muscular dystrophies—triggered at different spatial and temporal points, yet converging on similar downstream effects—one might expect a greater overlap in the splicing profiles of these myopathies. Addressing these questions will be crucial for future research.

## 6. Limitations

The limited number of samples is a possible limitation. However, due to the fact that these are rare diseases, there are no bigger numbers available. Another possible limitation is the slightly different origins of the materials; while the DM1 samples originate from tibialis anterior muscle biopsies, the FSHD samples are from quadriceps muscle biopsies, and the EDMD samples from primary muscle cell cultures differentiated in vitro to multinucleated myotubes. However, for all samples, the respective controls used for comparison are from the same origin.

## Data Availability

The original contributions presented in this study are included in the article/Supplementary Materials. Further inquiries can be directed to the corresponding author.

## Supplementary Materials

The following supporting information can be downloaded at https://www.mdpi.com/article/10.3390/biomedicines13030606/s1. Supplementary Table S1: List of splicing factors; Supplementary Table S2: DESeq2 analysis of splicing factors; Supplementary Table S3: Majiq analysis of misspliced genes; Supplementary Table S4: Majiq analysis of missplicing events, DM1; Supplementary Table S5: Majiq analysis of missplicing events, EDMD; Supplementary Table S6: Majiq analysis of missplicing events, FSHD.

## Abbreviations

The following abbreviations are used in this manuscript:

DMD	Duchenne muscular dystrophy
DMPK	dystrophia myotonia protein kinase
DM1	myotonic dystrophy type 1
EDMD	Emery–Dreifuss muscular dystrophy
FSHD	facioscapulohumeral muscular dystrophy
LSV	local splicing variation
MBNL	muscle-blind protein
NE	nuclear envelope
psi	percent spliced in
RAN translation	repeat-associated non-ATG translation
